# Hippuric Acid Suppresses Triple-Negative Breast Cancer via the EGFL8-Notch Signaling Axis

**DOI:** 10.3390/biomedicines14061400

**Published:** 2026-06-21

**Authors:** Tianhan Xu, Saijun Wang, Shuai Chen, Yan Zhou, Dongmei Wang

**Affiliations:** Department of Gastrointestinal Surgery, Changzhou Medical Center, The Third Affiliated Hospital of Nanjing Medical University, The Affiliated Changzhou No.2 People’s Hospital of Nanjing Medical University, Nanjing Medical University, 68 Middle Gehu Road, Changzhou 213000, China; catherine_xth@163.com (T.X.); wangsaijun1212@163.com (S.W.); chenshuai96432213@163.com (S.C.); zhouyan@njmu.edu.cn (Y.Z.)

**Keywords:** hippuric acid, metabolomics, triple-negative breast cancer, EGFL8, Notch signaling

## Abstract

**Background/Objectives:** Triple-negative breast cancer (TNBC) remains a highly aggressive malignancy with limited therapeutic options due to the absence of well-defined molecular targets. Diet-induced obesity (DIO) promotes TNBC progression by reshaping systemic metabolism and inflammatory responses; however, the key circulating metabolites involved and their mechanisms remain largely unclear. This study aimed to identify key metabolites associated with TNBC progression and further investigate their biological functions and molecular mechanisms. **Methods:** Targeted metabolomics profiling was performed on serum samples from MMTV-PyMT spontaneous breast cancer mice to identify differential metabolites associated with DIO. Functional assays were conducted to evaluate the effects of hippuric acid on TNBC cell proliferation, migration, and invasion. RNA sequencing was conducted to explore downstream regulatory pathways, followed by validation of candidate targets using gain- and loss-of-function approaches as well as rescue experiments. **Results:** Hippuric acid was identified as a significantly altered metabolite in DIO conditions. Functional studies demonstrated that hippuric acid markedly inhibited the proliferation, migration, and invasion of TNBC cells, with minimal effects on non-TNBC cells. Transcriptomic analysis identified EGFL8 as a potential downstream target, which was further confirmed by qPCR and functional assays. Overexpression of EGFL8 suppressed malignant phenotypes, whereas its knockdown promoted tumor progression. Rescue experiments showed that EGFL8 partially mitigated the inhibitory effects of hippuric acid on TNBC, suggesting that it functions as an important mediator in this process. Mechanistically, hippuric acid exerted its anti-tumor effects at least in part through modulation of the EGFL8-Notch signaling axis. **Conclusions:** Hippuric acid suppresses TNBC progression via the EGFL8-Notch signaling pathway. These findings highlight a previously unrecognized role of a gut microbiota-derived metabolite in TNBC and suggest its potential as a therapeutic candidate, providing new prospective targets and a theoretical basis for metabolic intervention for TNBC.

## 1. Introduction

According to the latest estimates from the International Agency for Research on Cancer (IARC), approximately 2.3 million women were diagnosed with breast cancer worldwide in 2022, making it the most commonly diagnosed cancer among women [1]. Furthermore, the global burden of breast cancer is expected to increase substantially, with annual cases and deaths projected to reach 3.2 million and 1.1 million, respectively, by 2050 [2].

Triple-negative breast cancer (TNBC) is a highly aggressive subtype of breast cancer characterized by the lack of expression of estrogen receptor (ER), progesterone receptor (PR), and HER2 [3]. It is characterized by rapid proliferation, high invasiveness, and strong metastatic potential, leading to poor clinical outcomes [4]. Owing to the lack of actionable molecular targets, TNBC remains largely refractory to endocrine and HER2-targeted therapies, leaving chemotherapy, radiotherapy, and surgery as the main treatment options [5]. Therefore, there is an urgent need to explore the molecular mechanisms underlying TNBC development and progression, and to discover more effective therapeutic targets and strategies.

Accumulating evidence indicates that diet-induced obesity (DIO) is closely associated with cancer development and progression and is considered an independent risk factor for multiple malignancies [6]. DIO promotes tumor progression through mechanisms such as chronic inflammation, hormonal dysregulation, and metabolic reprogramming [7]. In addition, obesity-induced alterations in systemic metabolism and gut microbiota composition further contribute to a tumor-promoting microenvironment [8,9]. However, the specific circulating metabolites mediating the link between DIO and TNBC progression remain poorly defined.

Metabolomics has emerged as a powerful approach for systematically profiling small-molecule metabolites and uncovering metabolic alterations in various diseases [10,11]. It has been widely applied to identify biomarkers and to elucidate metabolic reprogramming in cancer [12,13]. In this study, targeted metabolomic analysis of serum from the FVB/N MMTV-PyMT spontaneous breast cancer mouse model, a well-established transgenic model of mammary tumorigenesis [14], identified hippuric acid as a metabolite significantly altered under DIO conditions. Hippuric acid, a gut microbiota–derived metabolite, has been implicated in the regulation of host metabolism and inflammatory responses [15,16]. Notably, reduced urinary levels of hippuric acid have been reported in patients with colorectal cancer, suggesting its potential role in tumor development and progression [17]. In addition, alterations in hippuric acid have been observed in metabolomic studies of several malignancies [18,19,20]. However, its role in breast cancer, particularly in TNBC, remains largely unexplored.

EGFL8, a member of the epidermal growth factor (EGF)-like protein family and a paralog of EGFL7, participates in biological processes such as cell adhesion, angiogenesis, and invasion and metastasis [21]. Although other members of the EGF-like protein family (such as EGFL7) have been extensively studied in tumors, the role of EGFL8 remains poorly characterized. Emerging evidence suggests that EGFL8 may contribute to tumor progression in several cancers, including hepatocellular carcinoma and ovarian cancer [22,23], and may also participate in regulating cell differentiation [24]. Nevertheless, its functional role and regulatory mechanisms in TNBC have not yet been systematically investigated.

Based on these observations, we hypothesize that hippuric acid acts as a tumor-suppressive metabolite in TNBC. In this study, we integrated metabolomic and transcriptomic analyses to identify hippuric acid and EGFL8 as potential key regulatory factors. We further investigated their functional roles and molecular mechanisms in TNBC, revealing a novel EGFL8–Notch signaling axis underlying the anti-tumor effects of hippuric acid in TNBC.

## 2. Materials and Methods

### 2.1. Reagents

DMEM, RPMI1640, FBS, 100 U/mL penicillin and 100 μg/mL streptomycin, and 0.25% trypsin were purchased from Thermo Scientific (Gibco, Grand Island, NY, USA). PBS (Cat#G4202-500ML) was purchased from Servicebio (Wuhan, China). Hippuric acid (HY-W016562) was purchased from MCE (Shanghai, China). CCK8 kit (Cat#C0038), Matrigel (Cat#C0383) and hematoxylin and eosin (Cat#C0105M) were purchased from Beyotime (Shanghai, China). 4% paraformaldehyde (Cat#BL539A) was purchased from Biosharp (Beijing, China). RNAiso Plus (Cat#9109) was purchased from Takara (Beijing, China). HiScript II Q RT SuperMix for qPCR (+gDNA wiper) (Cat#R223-01) and SYBR Green qPCR Master Mix (Cat#Q312-02) were purchased from Vazyme (Nanjing, China). Puromycin (Cat#540411) was purchased from Sigma (St Louis, MO, USA).

### 2.2. Analysis of Public Database

TCGA-BRCA transcriptomic and corresponding clinical data were obtained from the Genomic Data Commons (GDC) portal (https://portal.gdc.cancer.gov, accessed 1 May 2025). TPM-formatted RNA-seq expression data generated using the STAR pipeline were extracted and transformed as log2(TPM + 1) for gene expression visualization and statistical analyses. Samples were restricted to female patients who had not received neoadjuvant treatment. EGFL8 expression across pathological stages and TNM classifications was analyzed using the Xiantao Academic platform. Expression differences between normal breast tissues and breast cancer samples, as well as among different breast cancer subtypes, were evaluated using the UALCAN (https://ualcan.path.uab.edu/, accessed 1 May 2025) web portal based on TCGA-BRCA datasets. Statistical comparisons were performed using the Kruskal-Wallis test, and the data were visualized using the ggplot2 (3.4.4) package.

### 2.3. Analysis of Metabolomics Analysis

Metabolomic data generated in this study were analyzed for differential metabolites. Metabolites meeting the criteria of fold change ≥ 2, *p* < 0.05, and variable importance in projection (VIP) > 1 were considered significantly altered metabolites. Heatmaps were generated for visualization of differential metabolite profiles. For heatmap visualization, metabolite abundance data were log2(value + 1)-transformed and normalized by row. Hierarchical clustering was performed using Euclidean distance. Values were capped at 3 and −3 for visualization purposes.

Metabolite classification and Kyoto Encyclopedia of Genes and Genomes (KEGG) pathway analysis. Identified metabolites were annotated using the Human Metabolome Database (HMDB) and KEGG database. Metabolite classification was performed based on HMDB annotations to characterize the chemical classes and functional properties of the detected metabolites. Functional annotation was subsequently conducted using the KEGG database. To investigate the biological relevance of metabolic alterations, differential metabolites were mapped to the KEGG PATHWAY database. KEGG pathway annotation was used to identify the major biochemical metabolic pathways and signaling pathways in which the metabolites participate, thereby facilitating the interpretation of potential biological functions associated with metabolic changes.

### 2.4. RNA Sequencing and Differential Expression Analysis

For transcriptomic analysis, 4T1 cells were treated with 200 μM hippuric acid for 24 h. Total RNA was extracted and subjected to RNA sequencing by OE Biotech (Shanghai, China). Raw sequencing reads were processed to remove low-quality reads and adapters before alignment to the mouse reference genome. Gene expression levels were quantified and differential expression analysis was performed by the sequencing provider. Genes with a fold change ≥ 1.5 and *p* < 0.05, after excluding genes with zero expression values, were considered differentially expressed. Candidate genes were further evaluated using GEPIA2, UALCAN, and Kaplan-Meier Plotter databases based on their expression patterns and prognostic relevance in breast cancer. Differentially expressed genes (DEGs) were visualized using a bidirectional bar plot to display upregulated and downregulated genes. The RNA-seq data generated in this study have been deposited in the GEO database under accession number GSE334662.

### 2.5. Serum Samples

Mouse serum used in this study was obtained from the animal model described previously [25]. Briefly, mice were randomly assigned to control and treatment groups. The models were established in eight-week-old MMTV-PyMT mice on an FVB/N background (GemPharmatech, Nanjing, China) under high-fat diet (HFD, DIO group, *n* = 4) or normal-chow diet (NCD, Control group, *n* = 4) conditions. At the time of sacrifice, all MMTV-PyMT mice had developed mammary tumors, and blood was collected for serum metabolomic analysis. The use of these serum samples was covered under the same animal ethics approval (Nanjing Medical University, Approval No. IACUC-2304021).

Principal component analysis (PCA) was performed using normalized serum metabolomic data from HFD- and NCD-fed MMTV-PyMT mice to assess overall metabolic differences between groups.

### 2.6. Cell Culture

4T1, MDA-MB-231, MCF10A, MCF7, and HEK293T cells were obtained from the American Type Culture Collection (Manassas, VA, USA). The murine 4T1 cell line was used alongside human breast cancer cell lines to validate the observed effects in a mouse-derived TNBC system. 4T1 and MCF7 cells were cultured in RPMI 1640 medium, while MDA-MB-231 and HEK293T cells were maintained in high-glucose Dulbecco’s Modified Eagle Medium (DMEM). MCF10A cells were cultured in DMEM supplemented with 20 ng/mL EGF, 100 ng/mL cholera toxin, 10 mg/mL insulin, and 0.5 mg/mL hydrocortisone. All culture media were supplemented with 10% fetal bovine serum (FBS), 100 U/mL penicillin, and 100 mg/mL streptomycin. Cells were maintained at 37 °C in a humidified incubator with 5% CO_2_ and subcultured at 90% confluence using 0.25% trypsin-EDTA.

### 2.7. CCK8 Assay

Cells in the logarithmic growth phase were harvested and digested with 0.25% trypsin-EDTA to prepare a single-cell suspension. Cells were seeded into 96-well plates at a density of 5 × 10^3^ cells per well, with 100 μL of culture medium per well. The cells were incubated overnight at 37 °C in a humidified incubator with 5% CO_2_ to allow adherence. After cell attachment, corresponding treatments were applied according to the experimental design. After 24 h, 10 μL of Cell Counting Kit-8 reagent (CCK-8, Beyotime, C0038) was added to each well, and the plate was gently tapped to mix. The 96-well plate was then incubated in the dark at 37 °C for 1–2 h. The absorbance (OD value) at 450 nm was measured using a microplate reader. Cell viability (%) was calculated using the formula: (OD value of experimental group − OD value of blank group)/(OD value of control group − OD value of blank group) × 100%. Each group included six replicate wells, and all experiments were independently repeated three times.

### 2.8. Transwell Assay

Cells in the logarithmic growth phase were digested with 0.25% trypsin-EDTA to obtain a single-cell suspension. The complete medium was discarded, and cells were resuspended in serum-free medium without antibiotics. After counting, the cell density was adjusted to 5 × 10^5^ cells/mL. Then, 200 μL of the cell suspension (approximately 1 × 10^5^ cells) was seeded into the upper chamber of a Transwell insert, while 600 μL of complete medium was added to the lower chamber. The Transwell chambers were incubated at 37 °C in a humidified incubator with 5% CO_2_ (16 h for migration assays and 24 h for invasion assays). After incubation, the upper chambers were fixed with 4% paraformaldehyde for 15 min, followed by hematoxylin and eosin (H&E, Beyotime, C0105M) staining according to the manufacturer’s instructions. Cells remaining on the upper surface of the membrane were gently removed with a cotton swab. After air-drying, five randomly selected fields were imaged using an inverted microscope (200× magnification) and cells were counted. Each group included three replicate wells, and all experiments were independently repeated three times. For the invasion assay, prior to cell seeding, 64 μL of Matrigel (Beyotime, C0383), diluted 1:8 in serum-free medium without antibiotics, was evenly coated onto the bottom of the upper chamber and incubated at 37 °C for 3 h to allow gel solidification. Subsequently, 100 μL of medium was added for hydration for 0.5 h. The remaining steps were the same as those used in the migration assay.

### 2.9. RNA Extraction and Quantitative PCR

Cells were treated with 200 μM hippuric acid for 24 h before RNA extraction. Total RNA was extracted from cells using TRIzol (TaKaRa) reagent according to the manufacturer’s instructions. Subsequently, 1 μg of total RNA was reverse-transcribed into cDNA using HiScript II Q RT SuperMix for qPCR (+gDNA wiper) (Vazyme, R223-01). Real-time quantitative PCR (qPCR) was then performed using ChamQ Blue Universal SYBR qPCR Master Mix (Vazyme, Q312-02). β-actin was used as the internal control, and the relative expression levels of target genes were calculated using the 2−^ΔΔCt^ method. The primer sequences used in this study are listed in Table A1.

### 2.10. Colony Formation Assay

Cells in the logarithmic growth phase were digested with 0.25% trypsin-EDTA to obtain a single-cell suspension, followed by cell counting. Cells were seeded into 6-well plates at a density of 500 cells per well, with 2 mL of culture medium and the corresponding drug treatments added to each well. The plate was gently shaken to ensure even cell distribution. Cells were incubated at 37 °C in a humidified incubator with 5% CO_2_ for 10–14 days, and the medium was refreshed every 2–3 days until visible colonies formed. The culture medium was then removed, and cells were gently washed twice with PBS. Cells were fixed with 4% paraformaldehyde for 15 min. After removing the fixative, each well was stained with 1 mL of 0.1% crystal violet solution at room temperature for 20 min. Excess dye was rinsed off with running water, and the plates were air-dried before imaging and colony counting. Images were captured using a digital camera (smartphone) under standardized lighting conditions to ensure consistency across groups.

### 2.11. Plasmid Construction

The coding sequence of EGFL8 (NM_030652.4) was obtained from the NCBI database. The full-length target gene was amplified using a high-fidelity DNA polymerase. The PCR products were purified by gel extraction and ligated into the pCDH-CMV-MCS-EF1-Puro vector, which had been digested with the corresponding restriction enzymes, using T4 DNA ligase. The ligation products were transformed into DH5α competent cells and plated on LB agar containing ampicillin (100 μg/mL), followed by overnight incubation at 37 °C. Single colonies were selected for expansion, and plasmid DNA was extracted for restriction enzyme digestion analysis and sequencing validation. Subsequently, the lentiviral expression plasmid was co-transfected into HEK293T cells together with the packaging plasmids psPAX2 and pMD2.G. At 72 h post-transfection, the viral supernatant was collected, filtered, and stored at −80 °C for further use. For gene knockdown, shRNA oligonucleotides targeting EGFL8 were cloned into the pLKO.1 vector. The oligonucleotide sequences were as follows: sense: 5′- CCGGCGCGCTCTGAAGCAGGAGATTCTCGAGAATCTCCTGCTT CAGAGCGCGTTTTTG-3′; antisense: 5′- AATTCAAAAACGCGCTCTGAAGCAGGAG ATTCTCGAGAATCTCCTGCTTCAGAGCGCG-3′. The procedures for ligation, transformation, plasmid identification, and sequencing were performed as described above for the overexpression constructs.

### 2.12. Statistical Analysis

For each experiment conducted, including more than 3 independent replications, all experimental data are presented as mean ± standard deviation (SD). Statistical analyses were performed using GraphPad Prism 10.0 (GraphPad, San Diego, CA, USA). Comparisons between two groups were conducted using an unpaired two-tailed t-test with Welch’s correction, while comparisons among multiple groups were performed using one-way analysis of variance (one-way ANOVA). A *p*-value < 0.05 was considered statistically significant. Levels of significance are indicated as follows: * *p* < 0.05, ** *p* < 0.01, *** *p* < 0.001, and n.s. (not significant).

## 3. Results

### 3.1. Metabolomic Analysis Identifies Hippuric Acid as a Key Metabolite Associated with Tumor Progression

**Figure 1:**
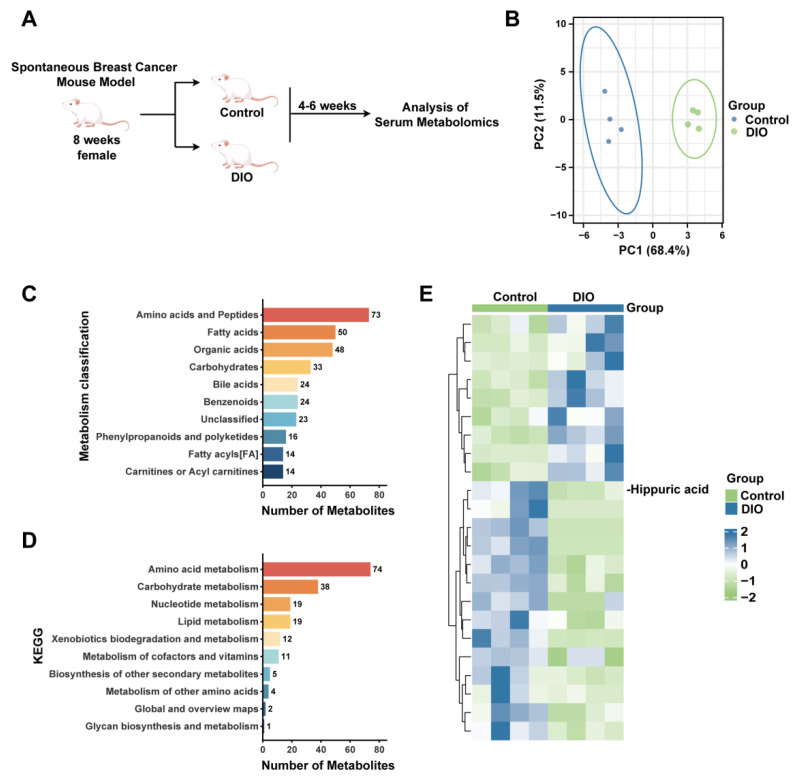
Impact of DIO on serum metabolomics in TNBC. (**A**) Schematic workflow of DIO-induced MMTV-PyMT mouse model. Created with Figdraw (https://www.figdraw.com). (**B**) PCA plot illustrating the clustering of biological samples (*n* = 4). PC1 and PC2 explained 68.4% and 11.5% of the total variance, respectively. (**C**) Bar chart depicting metabolite classification. Vertical axis: metabolism classification; Horizontal axis: number of metabolites. (**D**) Bar chart summarizing metabolic pathway classification. Vertical axis: KEGG pathway categories; Horizontal axis: number of metabolites. (**E**) Heatmap of differential metabolites between Control and DIO groups (Fold change ≥ 2, *p* < 0.05, VIP > 1). Data were log2(value + 1)-transformed and clustered using Euclidean distance after row normalization.

Building on our previous findings that a high-fat diet promotes tumor metastasis in the MMTV-PyMT spontaneous breast cancer mouse model [25], we further investigated circulating metabolic alterations to identify key metabolites associated with tumor progression. Targeted metabolomic profiling was performed on serum samples from the same cohort of mice (Figure 1A). Principal component analysis (PCA) revealed a clear separation between the DIO group and the normal control group, indicating a significant metabolic shift induced by high-fat feeding (Figure 1B). Among the 395 detected metabolites, the major classes included amino acids and peptides, fatty acids, organic acids, carbohydrates, bile acids, and aromatic compounds (Figure 1C). KEGG pathway enrichment analysis showed that these metabolites were mainly involved in amino acid metabolism, carbohydrate metabolism, nucleotide metabolism, lipid metabolism, and xenobiotic biodegradation and metabolism (Figure 1D). Based on predefined screening criteria, 23 metabolites were identified as significantly altered, including 14 upregulated and 9 downregulated metabolites (Figure 1E). The complete list of significantly altered metabolites is provided in Table A2. Notably, hippuric acid was significantly enriched in the normal diet group. As an endogenous metabolite jointly produced by the gut microbiota and host, hippuric acid is known to possess anti-inflammatory and antioxidant properties. Previous studies have reported reduced levels of hippuric acid in multiple cancers, suggesting a potential tumor-suppressive role [17,18,19,20]. Therefore, hippuric acid was selected for subsequent functional and mechanistic investigations.

### 3.2. Hippuric Acid Selectively Inhibits Proliferation, Migration, and Invasion of Triple-Negative Breast Cancer (TNBC) Cells

**Figure 2:**
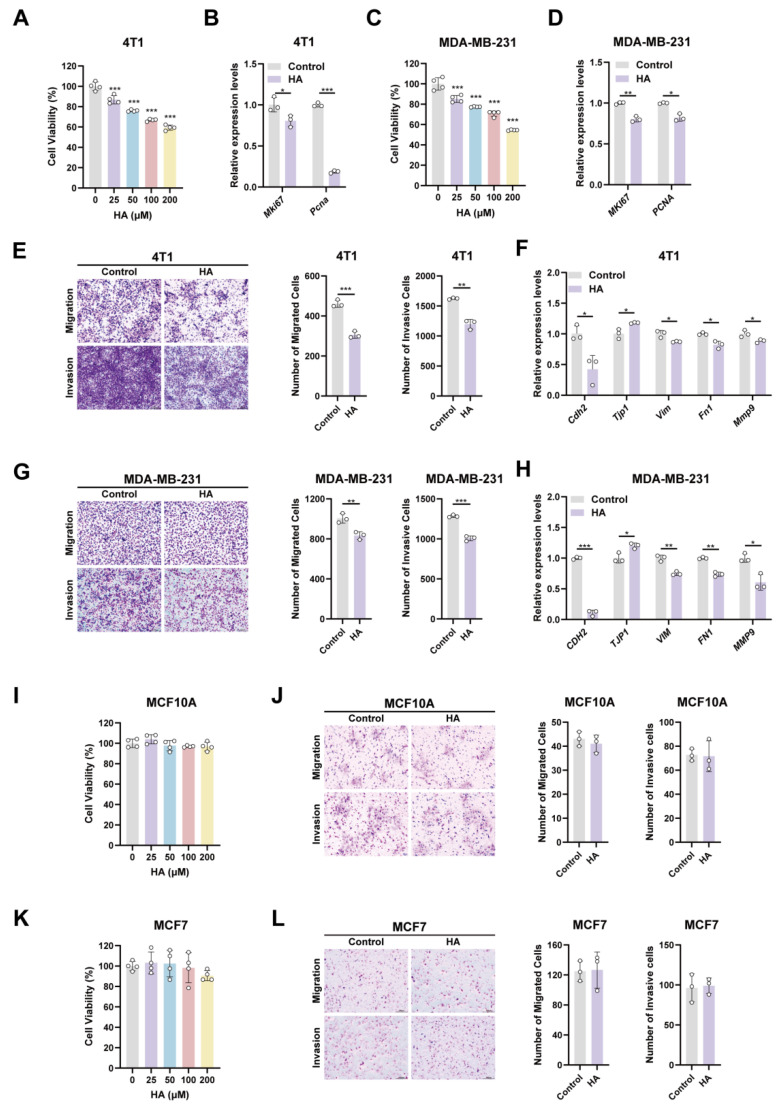
Hippuric acid (HA) suppresses proliferation, migration and invasion of TNBC cells. (**A**) CCK8 assay assessing cell proliferation of 4T1 cells treated with HA. (**B**) qRT-PCR analysis of proliferation-related genes (Ki67, Pcna) expression in 4T1 cells with HA. (**C**) Dose-dependent effects of HA on proliferation of MDA-MB-231 cells determined by CCK8 assays. (**D**) Relative mRNA expression levels of proliferative genes (Cdh2, Tjp1, Vim, Fn1 and Mmp9) in MDA-MB-231 cells determined by qRT-PCR. (**E**) Transwell assays demonstrated the effects of HA on migration and invasion of 4T1 cells. (**F**) Relative mRNA expression levels of EMT-related genes in 4T1 cells determined by qRT-PCR. (**G**) Transwell assays illustrating inhibition of migration and invasion by HA in MDA-MB-231 cells. (**H**) Relative mRNA expression levels of EMT-related genes in MDA-MB-231 cells determined by qRT-PCR. (**I**–**L**), The CCK8 assays and transwell assays in MCF10A (**I**,**J**) and MCF7 (**K**,**L**) cells treated with HA for 24 h.* *p* < 0.05; ** *p* < 0.01, *** *p* < 0.001.

Given the highly aggressive nature of TNBC, we next assessed the functional effects of hippuric acid on tumor cell behavior. CCK-8 assay demonstrated that hippuric acid significantly inhibited the proliferation of murine TNBC 4T1 cells in a dose-dependent manner (Figure 2A). Consistently, the expression of proliferation markers Ki67 and Pcna was markedly reduced (Figure 2B). Similar inhibitory effects were observed in the human TNBC cell line MDA-MB-231 (Figure 2C,D). Next, Transwell assays further revealed that hippuric acid significantly inhibited the migration and invasion capacities of 4T1 cells (Figure 2E). At the molecular level, hippuric acid downregulated mesenchymal markers (Cdh2, Vim, Fn1) and the invasion-related enzyme Mmp9, while upregulating the epithelial marker Tjp1 (Figure 2F), indicating inhibition of epithelial–mesenchymal transition (EMT). Comparable results were obtained in MDA-MB-231 cells (Figure 2G,H). To further evaluate the cell-type specificity of hippuric acid, we assessed its effects in normal mammary epithelial cells (MCF10A) and luminal A breast cancer cells (MCF7). Importantly, hippuric acid exhibited minimal effects on non-TNBC cells. Neither proliferation nor migratory and invasive capacities were significantly altered in normal mammary epithelial cells (MCF10A) or luminal A breast cancer cells (MCF7) (Figure 2I–L). Taken together, these results demonstrate that hippuric acid selectively suppresses the malignant phenotype of TNBC cells.

### 3.3. EGFL8 Is Identified as a Potential Downstream Mediator of Hippuric Acid

**Figure 3:**
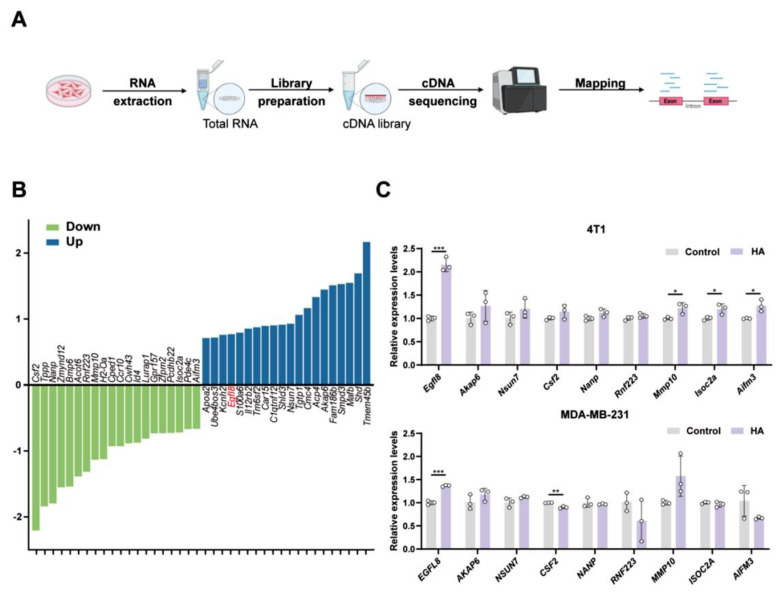
Transcriptome sequencing screens for downstream target genes. (**A**) Schematic depicting RNA-seq analysis of HA-treated 4T1 cells. Created in BioRender. Xu, C. (2026) https://BioRender.com/mpwnysp (**B**) Bidirectional bar plot showing the top 20 upregulated and downregulated genes in HA-treated TNBC cells compared to control. Blue bars represent upregulated genes, and green bars represent downregulated genes (Fold change ≥ 1.5, *p* < 0.05, excluding genes with zero expression value). (**C**) The mRNA expression levels of 9 candidate DEGs in 4T1 and MDA-MB-231 cells. * *p* < 0.05; ** *p* < 0.01, *** *p* < 0.001.

To elucidate the molecular mechanism underlying the anti-tumor effects of hippuric acid, RNA sequencing (RNA-seq) was performed on 4T1 cells, a murine TNBC cell line selected to maintain consistency with the mouse-derived metabolomic and in vivo experimental models, following treatment with hippuric acid (Figure 3A). A total of 54 differentially expressed genes (DEGs) were identified, including 31 upregulated and 23 downregulated genes. Based on fold change and statistical significance, the top 20 DEGs were selected. Combined with public databases analyses, nine candidate genes were further validated by qPCR, including Akap6, Nsun7, Egfl8, Csf2, Nanp, Rnf223, Mmp10, Isoc2a, and Aifm3 (Figure 3B). Among these, EGFL8 showed the most pronounced and consistent upregulation in both 4T1 and MDA-MB-231 cells, consistent with the RNA-seq results (Figure 3C). Given previous reports suggesting a role for EGFL8 in tumor progression [22,23,24], these data indicate that EGFL8 may serve as a potential downstream target mediating the inhibitory effect of hippuric acid on TNBC progression.

### 3.4. EGFL8 Suppresses TNBC Cell Proliferation, Migration, and Invasion

**Figure 4:**
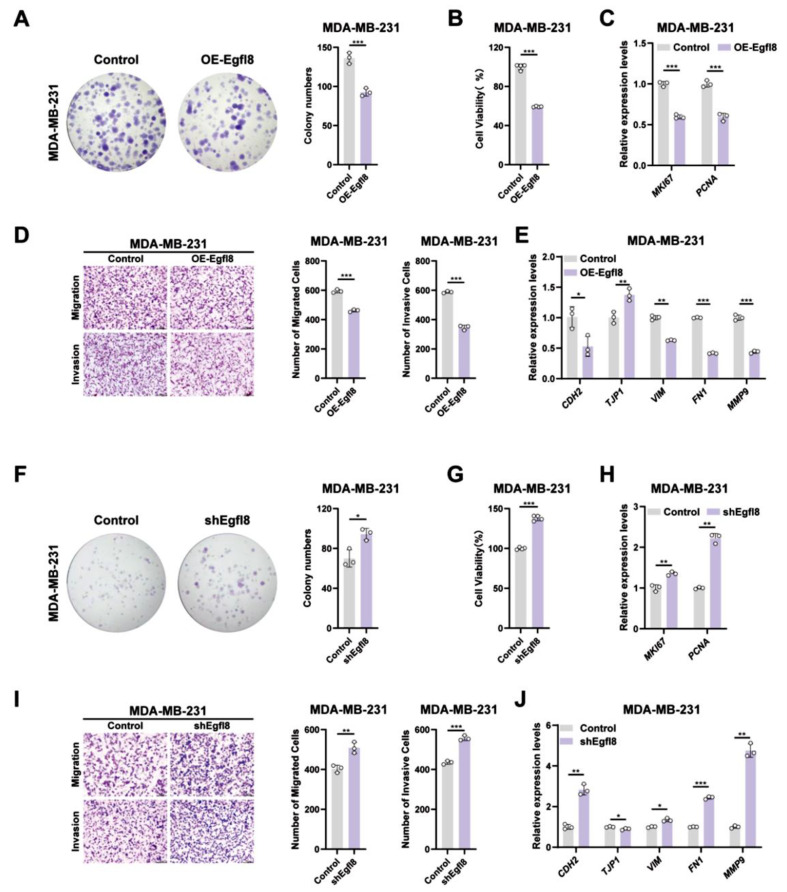
EGFL8 suppresses malignant phenotypes of TNBC cells. (**A**) Representative colony formation images (left) and quantification (right) in EGFL8-overexpressing MDA-MB-231 cells. (**B**) CCK8 assays of proliferation in EGFL8-overexpressing MDA-MB-231 cells. (**C**) qRT-PCR analysis of proliferation-related gene expression in EGFL8-overexpressing MDA-MB-231 cells. (**D**) Transwell migration and invasion assays in EGFL8 overexpressed MDA-MB-231 cells. (**E**) Relative mRNA expression of EMT-related genes in EGFL8 overexpressed MDA-MB-231 cells determined by qRT-PCR. (**F**) Representative colony formation images (left) and quantification (right) in EGFL8-knockdown MDA-MB-231 cells. (**G**,**H**) The CCK8 assay (**G**) and qRT-PCT analysis (**H**) of proliferative genes in EGFL8-knockdown MDA-MB-231 cells. (**I**) Representative images of the transwell migration and invasion assays in EGFL8 knockdown MDA-MB-231 cells. (**J**) Relative mRNA expression of EMT-related genes in EGFL8 knockdown MDA-MB-231 cells determined by qRT-PCR. * *p* < 0.05; ** *p* < 0.01, *** *p* < 0.001.

To determine the functional role of EGFL8, gain- and loss-of-function experiments were performed in MDA-MB-231 cells. The efficiency of EGFL8 overexpression and knockdown was confirmed by qRT-PCR (Supplementary Figure S1). EGFL8 overexpression significantly reduced the colony formation and proliferation, accompanied by downregulation of proliferation-related genes (Figure 4A–C). In addition, EGFL8 overexpression significantly inhibited migration and invasion, along with corresponding changes in EMT-related genes (Figure 4D,E). Conversely, EGFL8 knockdown promoted clonogenicity and proliferation (Figure 4F–H) and promoted migration and invasion, accompanied by EMT activation (Figure 4I,J). Taken together, these results establish EGFL8 as a tumor-suppressor in TNBC, with effects highly consistent with those induced by hippuric acid.

### 3.5. EGFL8 Mediates the Anti-Tumor Effects of Hippuric Acid

**Figure 5:**
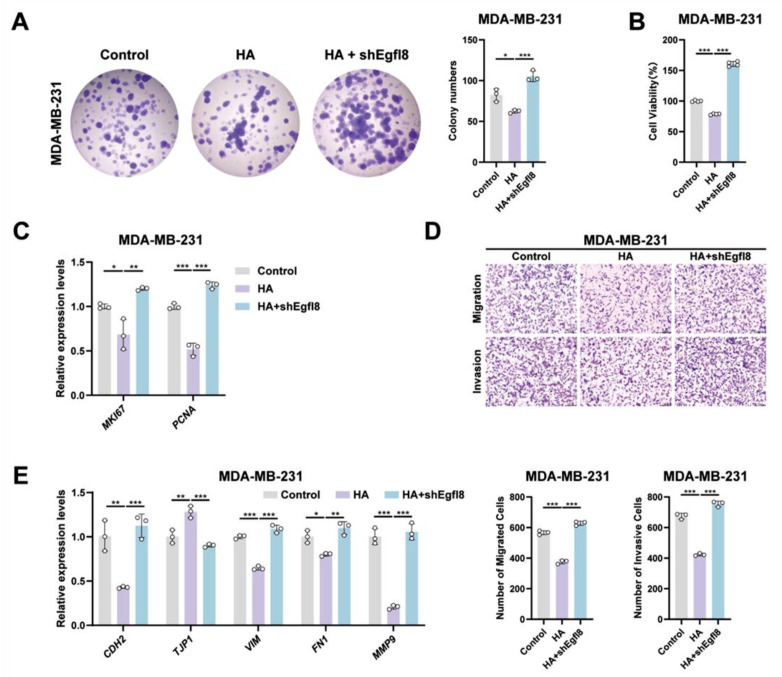
EGFL8 knockdown rescues HA-induced inhibition of MDA-MB-231 cell migration. (**A**) Representative images (left) and summary (right) of the colony formation assay for EGFL8-knockdown MDA-MB-231 cells treated with HA. (**B**) CCK8 assay in EGFL8-knockdown MDA-MB-231 cells with HA. (**C**) qRT-PCT analysis of proliferative genes in EGFL8-knockdown MDA-MB-231 cells treated with HA. (**D**) Transwell assays showing that EGFL8 knockdown rescues HA-mediated inhibition of migration and invasion in MDA-MB-231 cells. (**E**) Relative mRNA expression levels of EMT-related genes in EGFL8 knockdown MDA-MB-231 cells treated with HA determined by qRT-PCR. * *p* < 0.05; ** *p* < 0.01, *** *p* < 0.001.

To validate the functional link between hippuric acid and EGFL8, rescue experiments were performed. EGFL8 knockdown significantly attenuated the inhibitory effect of hippuric acid on colony formation and proliferation (Figure 5A,B), as well as on proliferation-related gene expression (Figure 5C). Similarly, the suppressive effects of hippuric acid on migration, invasion and EMT-related gene expression were partially reversed upon EGFL8 silencing (Figure 5D,E). Collectively, these findings indicate that EGFL8 is a critical mediator of the anti-tumor activity of hippuric acid.

### 3.6. The EGFL8/Notch Signaling Axis Is Involved in Hippuric Acid-Mediated TNBC Suppression

**Figure 6:**
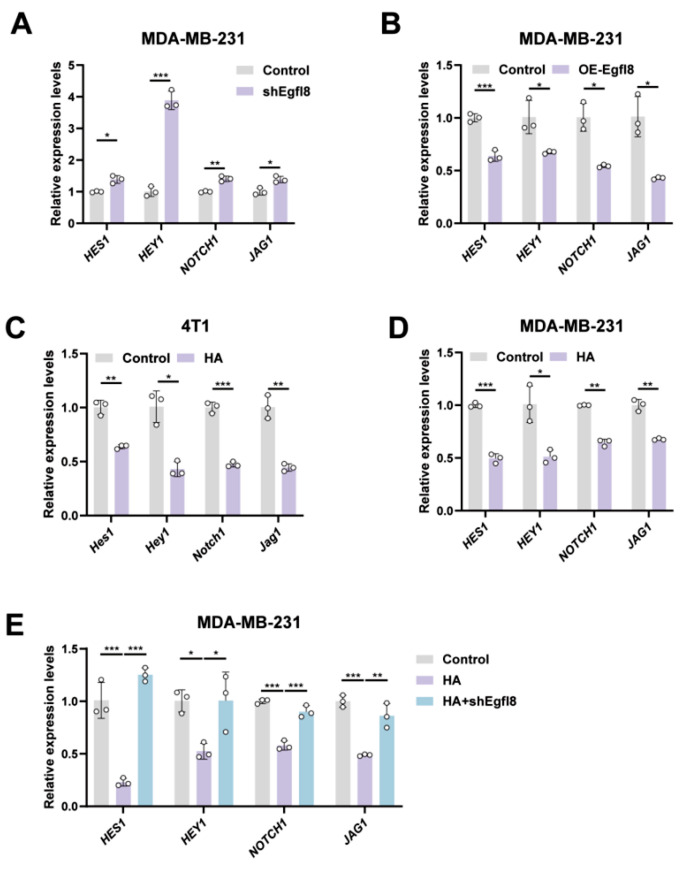
EGFL8 mediates the inhibitory effect of hippuric acid on the Notch signaling pathway. (**A**) qRT-PCR analysis of Notch pathway-related genes (Hes1, Hey1, Notch1 and Jag1) in MDA-MB-231 cells following EGFL8 knockdown. (**B**) Relative mRNA expression of Notch pathway-related genes in EGFL8 knockdown MDA-MB-231 cells determined by qRT-PCR. (**C**,**D**) qRT-PCR analysis of Notch pathway-related genes in 4T1 (**C**) and MDA-MB-231 (**D**) cells treated with HA. (**E**) Relative mRNA expression levels of Notch pathway-related genes in EGFL8 knockdown MDA-MB-231 cells treated with HA determined by qRT-PCR. * *p* < 0.05; ** *p* < 0.01, *** *p* < 0.001.

Based on previous studies reporting a potential regulatory relationship between EGFL8 and Notch signaling in cancer progression, we further investigated whether EGFL8 modulates this pathway [26]. EGFL8 knockdown significantly increased the expression of Notch1, Jag1, and downstream target genes Hes1 and Hey1 (Figure 6A), whereas EGFL8 overexpression produced the opposite effect (Figure 6B). Subsequently, we further explored the effects of hippuric acid on Notch signaling. Consistently, hippuric acid alone reduced the expression of Notch1 pathway components in both 4T1 and MDA-MB-231 cells (Figure 6C,D). Furthermore, hippuric acid treatment attenuated the EGFL8 knockdown–induced upregulation of these genes (Figure 6E), suggesting that EGFL8 may negatively regulate Notch-related molecules. These results suggest that hippuric acid suppresses TNBC progression, at least in part, through the EGFL8-Notch signaling pathway.

### 3.7. Clinical Relevance of EGFL8 in Breast Cancer

**Figure 7:**
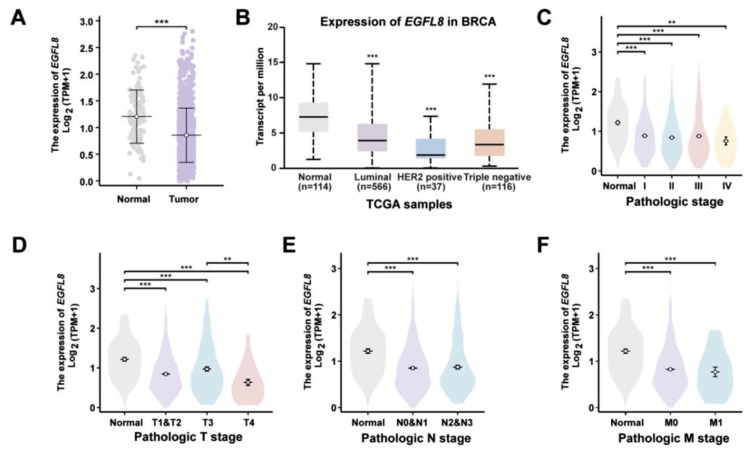
Clinical relevance of EGFL8 expression in breast cancer. (**A**) Comparison of EGFL8 expression between normal and breast cancer tissues. (**B**) Expression of EGFL8 across different breast cancer subtypes based on the TCGA database. (**C**) Correlation between EGFL8 expression and pathological stage. (**D**–**F**) Correlation between EGFL8 expression and TNM staging, including tumor size (**D**), lymph node involvement (**E**), and distant metastasis (**F**). ** *p* < 0.01, *** *p* < 0.001.

Analysis of public databases revealed that EGFL8 expression was significantly reduced in breast cancer tissues compared with normal tissues (Figure 7A). Subtype analysis further demonstrated differential EGFL8 expression among breast cancer subtypes, with notably reduced expression observed in TNBC (Figure 7B). In addition, EGFL8 expression decreased with advancing tumor stage (Figure 7C) and was significantly associated with T, N, and M classifications (Figure 7D–F), indicating a strong correlation with tumor progression and metastasis.

## 4. Discussion

In this study, we identified hippuric acid as a key metabolite associated with TNBC progression under diet-induced obesity conditions. Functional analyses demonstrated that hippuric acid selectively suppresses proliferation, migration, and invasion of TNBC cells, with minimal effects on non-TNBC cells, indicating a degree of tumor specificity.

Mechanistically, we identified EGFL8 as a key downstream mediator of hippuric acid. EGFL8 overexpression recapitulated the anti-tumor effects of hippuric acid, whereas its knockdown attenuated these effects, establishing a functional link between hippuric acid and EGFL8. Furthermore, we demonstrated that EGFL8 negatively regulates the Notch signaling pathway, providing a mechanistic basis for the observed phenotypes.

Hippuric acid is a typical gut microbiota–host co-metabolite formed through the conjugation of microbial-derived benzoic acid with host glycine metabolism [27], and its production depends on both gut microbial metabolism and hepatic conjugation processes [28,29]. Previous studies have reported that hippuric acid participates in multiple biological processes, including oxidative stress regulation [30], modulation of inflammatory and immune responses [15,16], and the serving as an important functional factor in regulating gut microbiota-disease interactions [31]. Although hippuric acid has long been investigated in various diseases, its direct role in cancer remains relatively understudied. Decreased urinary hippuric acid levels have been reported in colorectal cancer patients [17,18]. Furthermore, reduced serum hippuric acid levels have been observed in radioiodine-refractory thyroid cancer compared with conventional thyroid cancer [19]. Interestingly, Hatae et al. reported that non-small cell lung cancer patients who responded better to anti-PD-1 therapy had higher hippuric acid levels [20]. However, whether hippuric acid exerts a direct anti-tumor effect remains largely unclear. Our findings provide the first evidence that hippuric acid acts as a bioactive metabolic regulator that directly suppresses TNBC progression, extending its role beyond that of a metabolic biomarker, indicating its pharmacological potential. Some natural compounds, represented by betulinic acid, have been shown to induce cell cycle arrest, apoptosis, and inhibition of angiogenesis in TNBC cells [32,33]. Similar to these compounds, hippuric acid exhibited inhibitory effects on TNBC cell growth in the present study. However, whether hippuric acid shares common molecular mechanisms with these agents or exerts distinct biological effects remains to be further investigated.

Epidermal growth factor-like domain-containing protein 8 (EGFL8) belongs to the epidermal growth factor-like domain protein family and plays important roles in regulating cell adhesion, migration, and tissue development [34,35]. However, its role in cancer has been rarely reported. In this study, we identified EGFL8 as a novel tumor suppressor in TNBC and uncovered its role in mediating metabolic signaling. Importantly, our data link EGFL8 to the Notch signaling pathway [26], a key regulator of tumor proliferation, survival, and metastasis. The Notch signaling pathway is a highly conserved intercellular communication system that promotes tumor cell proliferation, survival, and anti-apoptotic capacity [36,37], as well as EMT, invasion, and metastasis [38,39]. In this study, key components of the Notch pathway showed consistent changes under EGFL8 modulation and hippuric acid treatment, suggesting that the EGFL8/Notch signaling axis contributes to hippuric acid-mediated suppression of TNBC, providing a mechanistic framework for its anti-tumor effects. Interestingly, hippuric acid demonstrated greater anti-tumor efficacy in TNBC cell lines than in MCF7 cells, despite the altered expression of EGFL8 across multiple breast cancer subtypes. This observation suggests that the biological effects of hippuric acid may be influenced by subtype-specific molecular contexts beyond EGFL8 expression alone. Although pan-Notch pathway inhibitors, such as γ-secretase inhibitors, have demonstrated anti-tumor activity, their clinical application has been limited by dose-limiting adverse effects, particularly gastrointestinal toxicity [40]. In contrast, as an endogenous metabolite, hippuric acid exhibited anti-tumor activity without apparent cytotoxic effects under the experimental conditions used in this study. Nevertheless, its efficacy and safety profile require further investigation in future preclinical and clinical studies.

Despite these findings, several limitations should be acknowledged. First, in vivo validation of hippuric acid in TNBC models is still required. Second, the upstream regulatory mechanism by which hippuric acid regulates EGFL8 expression remains unclear. Third, further validation at the protein and functional levels is needed to fully establish Notch pathway involvement. Finally, the clinical relevance of our findings requires further investigation, including whether hippuric acid levels are associated with breast cancer risk or prognosis in human cohorts, and whether the EGFL8/Notch axis plays a similar role in human TNBC.

## 5. Conclusions

In summary, this study demonstrates that hippuric acid suppresses TNBC progression by upregulating EGFL8 and inhibiting the Notch signaling pathway. These findings uncover a novel gut microbiota–metabolite–tumor signaling axis and highlight hippuric acid as a potential therapeutic candidate. Future studies integrating in vivo models and clinical validation will be essential to further evaluate the translational potential of targeting the hippuric acid–EGFL8–Notch axis in TNBC.

## Data Availability

The original contributions presented in this study are included in the article and Supplementary Materials. Further inquiries can be directed to the corresponding author.

## Supplementary Materials

The following supporting information can be downloaded at: https://www.mdpi.com/article/10.3390/biomedicines14061400/s1, Supplementary Figure S1: Validation of EGFL8 overexpression and knockdown efficiency.

## Abbreviations

The following abbreviations are used in this manuscript:

TNBC	Triple-negative breast cancer
DIO	Diet-induced obesity
MMTV-PyMT	Mouse Mammary Tumor Virus–Polyoma Middle T-antigen
ER	Estrogen receptor
PR	Progesterone receptor
EGFL8	Epidermal growth factor-like domain-containing protein 8
EGF	Epidermal growth factor
PCA	Principal component analysis
KEGG	Kyoto encyclopedia of genes and genomes
EMT	Epithelial–mesenchymal transition
DEGs	differentially expressed genes
VIP	Variable importance in projection
HA	Hippuric acid

## Appendix A

**Table A1:** Primers used for plasmid constructions and real-time PCR.

Gene Names	Forward 5′-3′	Reverse 5′-3′
hKi67 (NM_001145966.2)	AGAAGAAGTGGTGCTTCGGAA	AGTTTGCGTGGCCTGTACTAA
hPcna (NM_002592.2)	CCTGCTGGGATATTAGCTCCA	CAGCGGTAGGTGTCGAAGC
hCdh2 (NM_001308176)	ATGCTGACGATCCCAATGC	GCCTTCCATGTCTGTAGCTTGA
hTjp1 (NM_003257.4)	CAACATACAGTGACGCTTCACA	CACTATTGACGTTTCCCCACTC
hVimentin (NM_003380.5)	GACGCCATCAACACCGAGTT	CTTTGTCGTTGGTTAGCTGGT
hFibronectin (NM_212482.3)	CGGTGGCTGTCAGTCAAAG	AAACCTCGGCTTCCTCCATAA
hMmp9 (NM_004994.3)	GGGACGCAGACATCGTCATC	TCGTCATCGTCGAAATGGGC
hActb (NM_001101.5)	CTGGCACCACACCTTCTACAATGAG	GATAGCACAGCCTGGATAGCAACG
hGapdh (NM_001256799)	CCACTGCCAACGTGTCAGTGGT	CCCAGCAGTGAGGGTCTCTCTCT
hEgfl8 (NM_030652.4)	TCTGCTCGCACCATTGTTTTA	ACGCTGAGTATGCTGGCAC
hAkap6 (NM_004274.5)	AGTTCTCCCTAAAGCTGCTGT	TCTGCCTAGTGTAGTTGCCATT
hNsun7 (NM_001330648.3)	GCATTGGCAAGATGTCGAATC	GGCCCTTAGTTCCTGTTTCCTA
hCsf2 (NM_000758.4)	TCCTGAACCTGAGTAGAGACAC	TGCTGCTTGTAGTGGCTGG
hNanp (NM_152667.3)	GTGAGCCGGTAGCTTCCTTT	GCCACGTGCCTTGCAATTAT
hRnf223 (NM_001205252.2)	TGATGCTCTTCTGTGTGGCA	TTATCAGTCAGAGGCCCGAG
hMmp10 (NM_002425.3)	TTTGGCTCATGCCTACCCAC	TCTTGCGAAAGGCGGAACTG
hIsoc2a (NM_001136201.2)	ATCCTCTGTCCTGTTCCTGTG	CTGAGACGATCTGTGGGAAGTA
hAifm3 (NM_001146288.2)	CTGTCTGCCACGTCAAGGAC	GCCGTAGTGCGGACACTTAT
hHes1 (NM_005524.4)	ATGACAGTGAAGCACCTCCG	CGTTCATGCACTCGCTGAAG
hHey1 (NM_001040708.2)	TGAGAAGCAGGTAATGGAGCAA	TAGTCCATAGCAAGGGCGTG
hNotch1 (NM_017617.5)	GCAAGAACGCCGGGACAT	GGTTGGTGAGGCAGGCATTG
hJag1 (NM_000214.3)	TGTGAGGCCAAACCTTGTGT	AGGCGTCATTCTGACACTGG
mKi67 (NM_001081117.2)	ATCATTGACCGCTCCTTTAGGT	GCTCGCCTTGATGGTTCCT
mPcna (NM_011045.2)	TTTGAGGCACGCCTGATCC	GGAGACGTGAGACGAGTCCAT
mCdh2 (NM_007664.5)	AGCGCAGTCTTACCGAAGG	TCGCTGCTTTCATACTGAACTTT
mTjp1 (NM_009386.2)	GCCGCTAAGAGCACAGCAA	TCCCCACTCTGAAAATGAGGA
mVimentin (NM_011701.4)	CGTCCACACGCACCTACAG	GGGGGATGAGGAATAGAGGCT
mFibronectin (NM_010233.2)	ATGTGGACCCCTCCTGATAGT	GCCCAGTGATTTCAGCAAAGG
mMmp9 (NM_013599.5)	CTGGACAGCCAGACACTAAAG	CTCGCGGCAAGTCTTCAGAG
mActb (NM_007393.5)	CACTGTCGAGTCGCGTCC	CGCAGCGATATCGTCATCCA
mGapdh (NM_001289726)	AGGTCGGTGTGAACGGATTTG	GGCCTCACCCCATTTGATGT
mEgfl8 (NM_152922.4)	AGTTTGGGAGTGTGCTCCAAG	CACGGTATGTGGTCCTGTAGG
mAkap6 (NM_198111.2)	GTGGATGTGCATCTAGTTCAGC	CATTGACCGAGTAGGACAGCA
mNsun7 (NM_001400914.1)	TGGACCCAACGAGTGAAAGG	GTATTGGCGACTACATCCCCC
mCsf2 (NM_009969.4)	GGCCTTGGAAGCATGTAGAGG	GGAGAACTCGTTAGAGACGACTT
mNanp (NM_026086.2)	GGACAACACACTCATCGACAC	AGCACTCCTTGCTCAGTTTAAC
mRnf223 (NM_001220499.3)	CTGCGTTGCATTAACCGACC	GGTGACCCTAGTTGTTGGCT
mMmp10 (NM_019471.3)	CGCTGAGAGGGGAAGTCCTA	AGAGTGGGCCAAAATGCTGA
mIsoc2a (NM_001101598.1)	AAGCCTGCATCTTGAACACAG	GCGAGGAAAGCTCCACTCTG
mAifm3 (NM_001291070.1)	ACCAACGTGCTCATAGTGGG	AGGACAATCCGGTCTGAGAAG
mHes1 (NM_008235.3)	CCAGCCAGTGTCAACACGA	AATGCCGGGAGCTATCTTTCT
mHey1 (NM_010423.2)	AGTTAACTCCTCCTTGCCCG	TGGTCTCGTCCAGCTCACTA
mNotch1 (NM_008714.3)	TGTGGCTTCCTTCTACTGCG	CTTTGCCGTTGACAGGGTTG
mJag1 (NM_013822.5)	GAGCGCCTCAAAGAAGCGA	ATTGGAGCATGCACGACTGG

**Table A2:** Differential serum metabolites identified between HFD- and NCD-fed MMTV-PyMT mice.

Metabolite	FC(NCD/HFD)	VIP	*p* Value
Guanosine monophosphate	0.03	1.15	0.002
Guanosine (G)	0.04	1.03	0.004
Tartaric acid	0.15	1.60	<0.001
3,7-Diketocholanic Acid	0.22	2.02	<0.001
2-Hydroxy-2-methylbutyric acid	0.33	1.48	0.006
Piceid	0.37	1.56	0.019
Glutarylcarnitine(C5DC)	0.42	1.63	0.003
Oxalacetic acid	0.43	1.51	0.006
Ribose 5-phosphate	0.47	1.26	0.028
Ursocholic Acid(UCA)	2.02	1.45	0.020
2-Deoxyglucose	2.26	1.52	0.013
Octanoylcarnitine(C8)	2.28	1.64	0.024
Xanthosine	2.43	1.67	0.002
23,24-bisnor-5-cholenic acid-3beta-OL acetate	2.63	1.50	0.015
4-androsten-17alpha-ol-3-one-17beta-carboxylic acid acetate	3.19	1.56	0.007
7-Ketolithocholic Acid Acetate Methyl Ester	3.83	1.37	0.015
4-Hydroxyphenyl-2-propionic acid	4.49	1.56	0.024
Hippuric acid	8.49	1.44	0.019
Trigonelline	8.97	2.045	0.042
Indole-3-propionic acid	9.50	1.59	<0.001
myo-Inositol	145,161.05	1.26	0.022
Indole-3-methyl acetate	7,875,433.71	1.58	0.004
L-Sorbose	9,819,533.37	1.34	0.021
