# Supplementary material for: Hippuric Acid Suppresses Triple-Negative Breast Cancer via the EGFL8-Notch Signaling Axis
Source: Biomedicines. 2026 Jun 21;14(6):1400. doi: 10.3390/biomedicines14061400 (PMC13297136; doi:10.3390/biomedicines14061400)
Supplement: Supplementary file 1 [file biomedicines-14-01400-s001.zip › biomedicines-4342687-supplementary.pdf]

biomedicines

MDPI

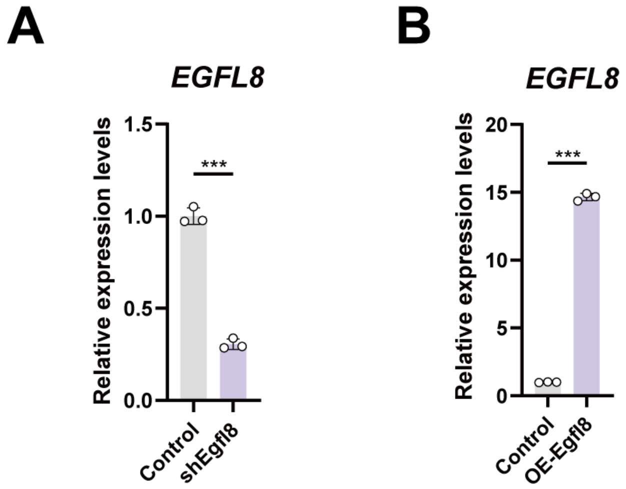

Supplementary Figure S1. Validation of EGFL8 overexpression and knockdown efficiency. (**A**) qRT-PCR analysis confirming the knockdown efficiency of EGFL8 in MDA-MB-231 cells transfected with shEGFL8 compared with control. (**B**) qRT-PCR analysis confirming the overexpression efficiency of EGFL8 in MDA-MB-231 cells transfected with EGFL8 expression plasmid compared with vector control. *$p < 0.05$; **$p < 0.01$, ***$p < 0.001$.
